# Utilizing plant synthetic biology to accelerate plant-microbe interactions research^☆^

**DOI:** 10.1016/j.bidere.2025.100007

**Published:** 2025-03-18

**Authors:** Xiaohan Yang, Joanna Tannous, Tomás A. Rush, Ilenne Del Valle, Shunyuan Xiao, Bal Maharjan, Yang Liu, David J. Weston, Kuntal De, Timothy J. Tschaplinski, Jun Hyung Lee, Mallory Morgan, Daniel Jacobson, Md Torikul Islam, Feng Chen, Paul E. Abraham, Gerald A. Tuskan, Mitchel J. Doktycz, Jin-Gui Chen

**Affiliations:** a Biosciences Division, Oak Ridge National Laboratory, Oak Ridge, TN, 37831, USA; b The Center for Bioenergy Innovation, Oak Ridge National Laboratory, Oak Ridge, TN, 37831, USA; c Institute of Biosciences and Biotechnology Research and Department of Plant Science and Landscape Architecture, University of Maryland, College Park, MD, 20850, USA; d Department of Plant Sciences, University of Tennessee, Knoxville, TN, 37996, USA

**Keywords:** Synthetic biology, Plant-microbe interactions, Symbiosis, Disease resistance, Genetic engineering

## Abstract

Plant-microbe interactions are critical to ecosystem resilience and substantially influence crop production. From the perspective of plant science, two important focus areas concerning plant-microbe interactions include: 1) understanding plant molecular mechanisms involved in plant-microbe interfaces and 2) engineering plants for increasing plant disease resistance or enhancing beneficial interactions with microbes to increase their resilience to biotic and abiotic stress conditions. Molecular biology and genetics approaches have been used to investigate the molecular mechanisms underlying plant responses to various beneficial and pathogenic microbes. While these approaches are valuable for elucidating the functions of individual genes and pathways, they fall short of unraveling the complex cross-talk across pathways or systems that plants employ to respond and adapt to environmental stresses. Also, genetic engineering of plants to increase disease resistance or enhance symbiosis with microbes has mainly been attempted or conducted through targeted manipulation of single genes/pathways of plants. Recent advancements in synthetic biology tool development are paving the way for multi-gene characterization and engineering in plants in relation to plant-microbe interactions. Here, we briefly summarize the current understanding of plant molecular pathways involved in plant interactions with beneficial and pathogenic microorganisms. Then, we highlight the progress in applying plant synthetic biology to elucidate the molecular basis of plant responses to microbes, enhance plant disease resistance, engineer synthetic symbiosis, and conduct *in situ* microbiome engineering. Lastly, we discuss the challenges, opportunities, and future directions for advancing plant-microbe interactions research using the capabilities of plant synthetic biology.

## Abbreviations

AMF: Arbuscular mycorrhizal fungi
CDPKs: Calcium-dependent protein kinases
EMF: Ectomycorrhizal fungi
ETI: Effector-triggered immunity
ETS: Effector-triggered susceptibility
HR: Hypersensitive response
ISR: Induced systemic resistance
LCOs: Lipo-chitooligosaccharides
MAMPs: Microbe-associated molecular patterns
MAPKs: Mitogen-activated protein kinases
NLR: Nucleotide-binding leucine-rich-repeat
PAMPs: Pathogen-associated molecular patterns
PRRs: Pattern recognition receptors
PTI: Pattern-triggered immunity
RLCKs: Receptor-like cytoplasmic kinases
RLK: Receptor like kinase
SAR: Systemic acquired resistance

## 1 Introduction

Plants coexist with, shape, and benefit from a dynamic community of microorganisms, collectively referred to as the microbiota. The microbiota encompasses fungi, oomycetes, protozoa, bacteria, archaea, nematodes, and viruses. The relationships between plants and their interacting microbes span a continuum, ranging from mutualistic (beneficial to both), commensal (beneficial to one while not harmful to the other), to pathogenic (harmful to the host). This intricate system of plant-microbe interactions plays a vital role in shaping plant growth, development, and adaptation to environmental stresses [[1], [2], [3], [4], [5]]. Beneficial interactions, such as those between plant roots and endophytic/mycorrhizal fungi or nitrogen-fixing bacteria, facilitate plant nutrient acquisition and enhance plant defense and abiotic stress tolerance [3,5]. Conversely, harmful interactions (e.g., plant infection with various pathogens) often lead to plant diseases that may cause severe crop losses or death [3,6].

Plants serve as both hosts and regulators of plant-associated microbial communities [7,8]. To better understand the intricate nature of the holobiont, which refers to the collective entity comprising the host organism and its associated microbiota, it is imperative to delve deeper into the molecular processes that govern the dynamic and complex interactions between plants and their associated microbes. While molecular biology and genetics approaches have been used to investigate such molecular mechanisms, these approaches are limited to discovering and characterizing functions of individual genes. However, the intricate nature of these interactions often involves the concerted action of multiple genes and their associated pathways, necessitating a more holistic approach to unravel the complexity of these processes. The advent of "multi-omics" technologies, encompassing genomics, lipidomics, transcriptomics, ionomics, metabolomics, and proteomics has enabled researchers to contextually understand plant responses to microbial interactions [9]. These high-throughput analyses, coupled with genome-wide association studies (GWAS) that explore the natural variation in plant-microbe associations, have identified a large number of candidate genes or gene modules in plants that potentially regulate and control the complex interplay between plants and their associated microbiome [3,8,10,11]. Experimental characterization of the candidate genes predicted by integrative analysis of omics and GWAS data is technically challenging. Another major research area concerning plant-microbe associations is to engineer resistance against pathogens and enhance symbiosis with beneficial microbes through ectopic overexpression and genetic modification of single natural genes identified in plants. There is a growing interest in gene stacking in plants to achieve enhanced disease resistance [12,13]. However, combining multiple genes into the same genetic background via conventional plant breeding techniques is inherently time-consuming and resource intensive [13]. While traditional breeding approaches have yielded substantial progress in enhancing plant disease resistance, the genetic improvement of plant-microbial symbiosis within host species poses a far greater challenge. This is primarily due to the limited characterization of the complex network of genes involved in determining the outcome and output as well as the specificity of broad and diverse plant-symbiont interactions.

The limitations and challenges in studying plant-microbe interactions may be overcome through plant synthetic biology or plant biosystems design. This emerging interdisciplinary field combines engineering principles, biology, and computational sciences to redesign existing plant biological pathways or design new biological components and systems in plants for beneficial purposes [14,15]. Through iterative Design-Build-Test-Learn cycles, plant synthetic biology offers great potential for facilitating plant science research in two aspects: 1) accelerating genetic improvement of plant traits to benefit ecosystem health and human society and 2) providing a means to systematically manipulate biological systems to test specific hypotheses about how these systems work and gain a deeper understanding of their underlying mechanisms [15,16]. For example, synthetic biology can be used to engineer the form and function of root systems which impact plant-microbe interactions [17,18].

In this review, we first summarize the current understanding of plant molecular pathways involved in interactions with beneficial and pathogenic microorganisms to provide a biological framework for applying plant synthetic biology in plant-microbe interactions research. Next, we highlight the advances in various synthetic biology applications, including 1) gaining a deeper understanding of molecular mechanisms underlying plant responses to microbes through pathway engineering and construction of plant-based biosensors, 2) enhancing plant disease resistance through engineering resistance genes or host susceptibility factors, 3) creating synthetic symbioses through engineering plants to produce signaling molecules for regulating gene expression in beneficial microbes and/or express synthetic cell-surface receptors for binding and recognizing ligands derived from beneficial microbes, as well as components required for signaling during mycorrhization or nodulation, and 4) *in situ* microbiome engineering through modifying root exudate profiles and altering root architecture. Finally, we discuss the challenges, opportunities, and future research directions for leveraging synthetic biology to transform the study of plant-microbe interactions.

## 2 Plant molecular pathways involved in plant-microbe interactions

Plants have evolved distinctive signaling and metabolic pathways to express and/or activate resistance against pathogens and establish symbioses with beneficial microbes. Notably, some signaling/regulatory components are shared between the disease resistance and symbiotic pathways.

### 2.1 Plant molecular pathways involved in plant disease resistance

**Fig. 1:**
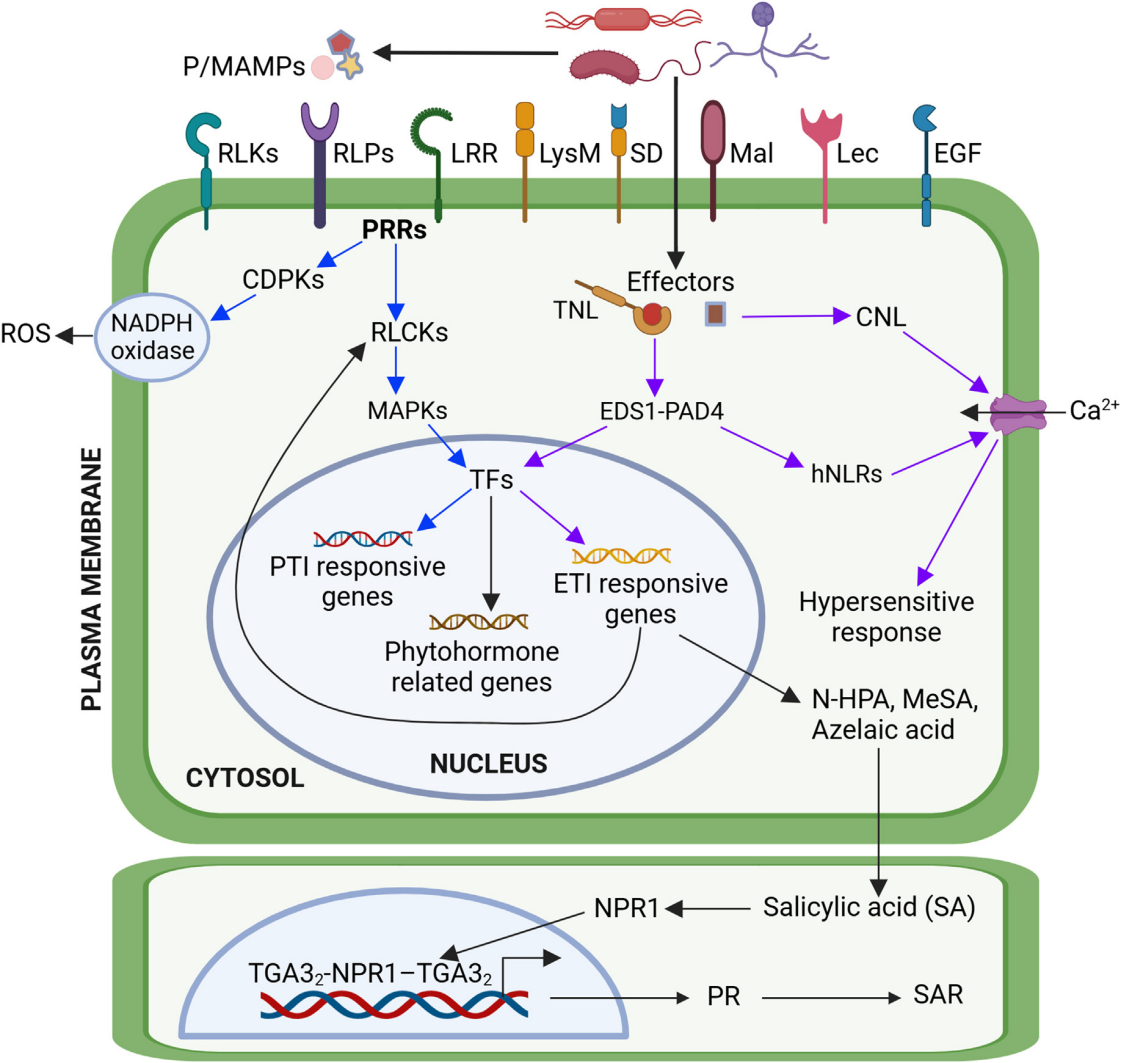
Molecular pathways involved in plant disease resistance. Pathogen/microbe-associated molecular patterns (P/MAMPs) are recognized by pattern recognition receptors (PRRs), such as receptor-like kinases (RLKs) and receptor-like proteins (RLPs), which have highly variable extracellular domains, including leucine-rich repeats (LRR), lysine motifs (LysM), lectin (Lec), S domain (SD), tandem malectin (Mal), and epidermal growth factor (EGF). PRRs activate pattern-triggered immunity (PTI) response (indicated by blue arrow) by engaging receptor-like cytoplasmic kinases (RLCKs), calcium-dependent protein kinases (CDPKs), and mitogen-activated protein kinases (MAPKs). This triggers an immune response in plants by activating transcription factors (TFs) that drive the expression of PTI-responsive genes. The effector-triggered immunity (ETI) pathway (indicated by the purple arrow) is activated when intracellular nucleotide-binding leucine-rich repeat receptors (NLRs) recognize pathogen-derived effectors. There are two types of sensor NLRs: Coiled-coil domain-containing NLR (CNL) resistosomes and Toll-interleukin 1-like receptor (TIR) NLR (TNL) resistosomes. Upon effector recognition, CNLs oligomerize and insert in the plasma membrane where they function as calcium channels, inducing calcium influx that triggers a hypersensitive response (HR). TNLs, upon effector-triggered oligomerization, act as NADases that generate small signaling molecules, activating downstream EDS1-PAD4 complexes, which, in turn, activate helper (RPW8) NLRs or RNLs. Oligomerized RNLs also insert into the plasma membrane, where their calcium channel activity induces defense signaling and hypersensitive response (HR). ETI results in programmed cell death (PCD) at the infection site and induces the production of mobile immune signals (e.g., N-hydroxypipecolic acid (N-HPA), methyl salicylic acid (MeSA), azelaic acid), which induce salicylic acid (SA) accumulation in nearby cells to activate nonexpressor of pathogenesis-related-1 (NPR1). NPR1 forms complex with TGA3 to drive the expression of pathogenesis related (PR) genes, resulting in systemic acquired resistance (SAR). Redrawn from Refs. [20,21,27,[35], [36], [37], [38]]. Created with BioRender.com.

Plant disease resistance relies on a complex immune system (Fig. 1) to recognize foreign (or non-self) molecules (e.g., pathogen ligands) via various plant receptors (either cell surface receptors, intracellular receptors, or both), carry out signal transduction, and respond defensively through pathways involving many genes and their products [[19], [20], [21]]. The plant immune system contains two interconnected branches: pattern-triggered immunity (PTI) and effector-triggered immunity (ETI) [[22], [23], [24]]. PTI is the first line of inducible defense, activated when pattern recognition receptors (PRRs) on the plant cell surface (i.e., the plasma membrane) recognize and bind to specific microbe- or pathogen-associated molecular patterns (MAMPS or PAMPS), such as chitin, flagellin, and peptidoglycan. Adapted pathogens, which have evolved specific mechanisms to successfully infect specific host species, secrete effectors into host cells and subvert PTI, resulting in effector-triggered susceptibility (ETS). ETI is the second line of inducible defense, activated by intracellular nucleotide-binding leucine-rich-repeat (NLR) proteins encoded by *R*-genes upon detection of the physical presence or virulence activity of effectors [21,25]. Thus, PTI and ETI are evolutionarily interrelated and mechanistically interconnected, with their signaling pathways converging at multiple nodes [26].

PRRs, including receptor-like kinases (RLKs, also known as receptor kinases) and receptor-like proteins (RLPs), employ highly variable extracellular domains to recognize a wide range of ligands, form complexes with co-receptors, which share similar extracellular domains, for ligand binding, and activate downstream receptor-like cytoplasmic kinases (RLCKs) to relay PTI signaling [20,27]. PTI also triggers signaling cascades involving the activation of calcium-dependent protein kinases (CDPKs) and mitogen-activated protein kinases (MAPKs), resulting in transcriptional reprogramming to activate defense responses [20,21].

ETI is more specifically activated upon infection by a pathogen expressing an effector that is recognized by a host R protein [28]. This recognition can occur through direct binding of the effector by the R protein or indirectly by the R protein surveilling for pathogen effectors binding to host proteins. The latter mechanism, often more robust, involves the R protein triggering a defense response when it recognizes a matching effector [29]. ETI often culminates with the hypersensitive response (HR) (i.e., a form of programmed cell death at the site of infection) to limit pathogen spread [20,30]. Additionally, ETI can induce the production of mobile signals, such as *N*-hydroxypipecolic acid [31], methyl salicylate (MeSA) [32], and azelaic acid [33], that are produced in and transported out from the infection site to uninfected tissues thereby inducing the accumulation of salicylic acid (SA) and mediating massive transcriptional programming, which results in systemic acquired resistance (SAR). Similar to local resistance, SAR is also associated with the production of pathogenesis-related (PR) proteins with antimicrobial activity that protects plants against attacks from a wide range of virulent pathogens [20,21]. SAR involves two sequence-related but functionally opposing SA co-receptors in transcriptional regulation of defense gene expression: the nonexpresser of PR 1 (NPR1) as a transcriptional co-activator and its homologs NPR3/4 as transcriptional co-repressors [20]. The SA-bound NPR1 dimer induces transcription by bridging two TGA transcription factor dimers, forming an enhanceosome (i.e., TGA3_2_–NPR1_2_–TGA3_2_) [30,34].

Although PTI and ETI are two distinct defense mechanisms with different amplitudes and dynamics, they are not mutually exclusive, and there is significant cross-talk between these two pathways [28]. Despite involving different early signaling components, PTI and ETI converge into many similar downstream responses, and mutually potentiate each other to achieve stronger plant defenses [37,39]. For example, NLRs play a key role in synchronizing PTI with ETI, thereby ensuring broad-spectrum resistance [40].

In addition to the plant's innate immunity system, beneficial members of the root microbiota can trigger so-called induced systemic resistance (ISR) in a wide range of plant hosts against various pathogens [41].

### 2.2 Plant molecular pathways involved in plant-microbe symbiosis

**Fig. 2:**
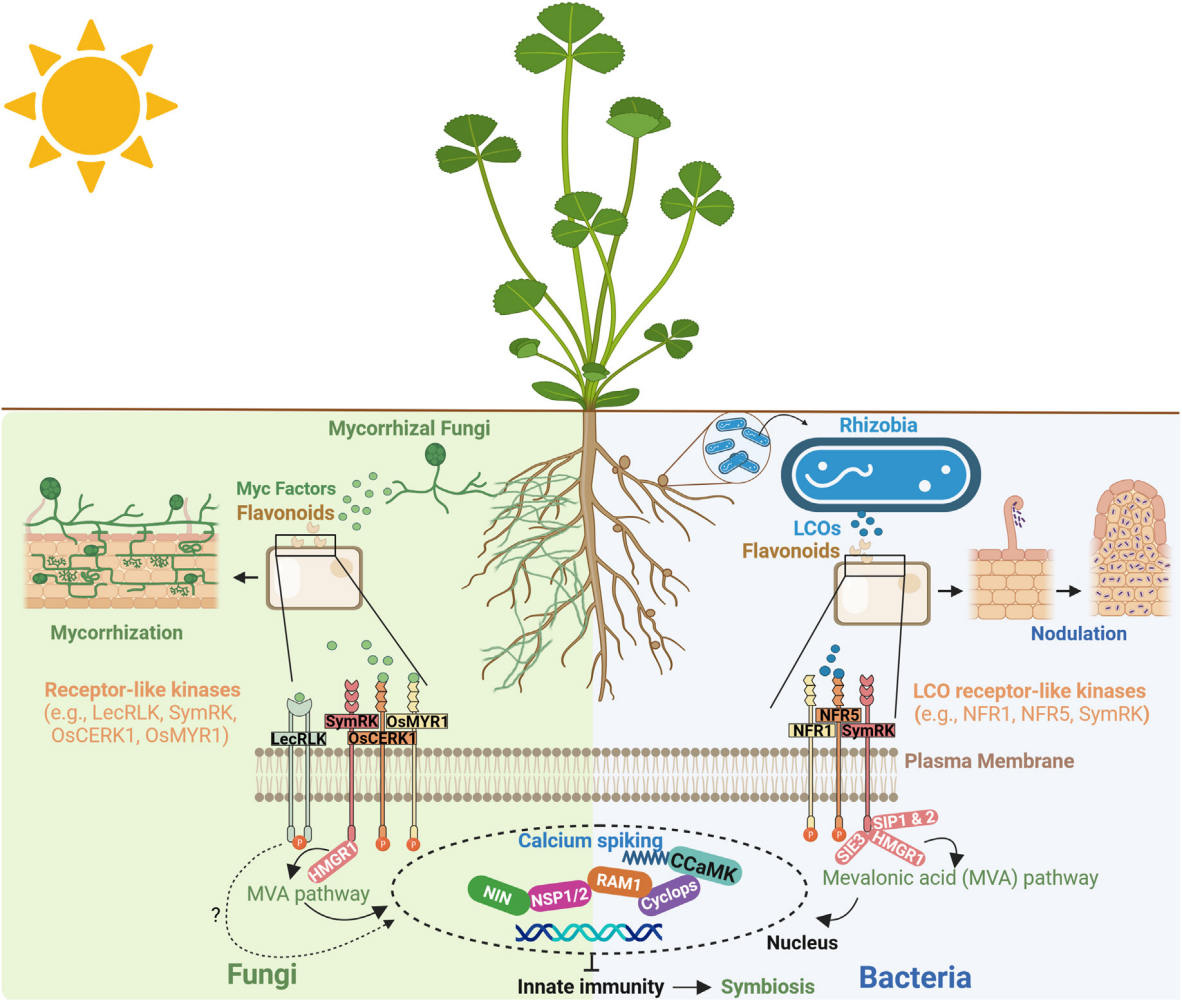
Molecular mechanisms mediating mycorrhizal and rhizobial symbioses. Plant roots secrete various flavonoids that are vital in establishing mutually beneficial relationships with rhizobial and mycorrhizal symbionts. Additionally, plants release other signaling molecules such as strigolactones (SL) and 2-hydroxy fatty acids, which play crucial roles in facilitating these symbiotic interactions. These signals control the expression of numerous microbial genes, including *nod* genes and stimulate fungal hyphal branching. They also stimulate the production of signaling molecules by the symbionts, such as rhizobial lipo-chitooligosaccharides (LCOs) (e.g. Nod factors) and mycorrhizal LCOs (e.g. Myc factors). These LCOs are detected by different receptor complexes. LCOs produced by rhizobia bacteria are perceived at the plasma membrane by receptor like kinases (RLKs) (e.g. NFR1 and NFR5). The perception of mycorrhizal LCOs also involve plant RLKs. LysM-RLKs are the most studied in symbioses with beneficial mycorrhizal fungi. Recently, a LysM-RLK receptor complex consisting of OsMYR1/OsLYK2 and OsCERK1 has been identified in rice mediating AMF perception [59]. These examples are highlighted because the key receptors for mycorrhizal LCOs have yet to be characterized in other plant species. In addition to LysM-RLKs, a G-type Lectin-RKs (LecRK) that mediates the symbiotic interaction between *Populus* and *Laccaria bicolor* has been reported. The downstream signaling cascades for lecRKs have not been extensively studied and remain largely unknown compared to LysM-RLKs. The signal emitted by LysM receptors activates the symbiosis receptor kinase (SymRK) that associates with essential proteins like SymRK interacting protein 1 and 2 (SIP1 and 2), SymRK-interacting E3 ligase (SIE3), and the 3-hydroxy-3-methylglutaryl coenzyme A reductase1 (HMGR1) involved in mevalonate biosynthesis, which triggers calcium oscillations in the nuclear region, also known as calcium spiking. Downstream of calcium spiking, a module involving calcium/CaM-dependent protein kinase (CCaMK) and Cyclops promotes mycorrhizal symbiosis by activating the expression of Reduced Arbuscular Mycorrhiza1 (RAM1) and promotes rhizobial symbiosis by forming a complex with Nodulation Signaling Pathways 1 and 2 (NSP1/NSP2) to regulate the expression of Nodule Inception (NIN). Created with BioRender.com.

The symbiotic relationship between plants and microbes involves various molecular pathways (Fig. 2) that enable plants to interact beneficially with microbes, such as the nodulation signaling pathway involved in the symbiosis between legumes and nitrogen-fixing bacteria (e.g., nodulating rhizobia), the mycorrhizal symbiosis pathways involved in the symbiosis between plants and mycorrhizal fungi, including arbuscular mycorrhizal fungi (AMF) and ectomycorrhizal fungi (EMF), or the signaling mechanisms used by plants such as the secretion of myo-inositol to attract specific bacterial species [11]. Many of these mechanisms on how microbial-encoded genes suppress plant molecular pathways have been thoroughly reviewed in Liu et al. [42].

In recent years, significant progress has been made towards understanding the molecular aspects of interactions between mycorrhizal fungi and plants. Recently, it has been discovered that these fungi release small proteins called mycorrhiza-induced small-secreted proteins (MiSSPs), which act as signals for symbiosis [43,44]. These proteins can either directly suppress defense signaling pathways in plants or indirectly regulate the expression of defense genes through host interactions. It has also been shown that plants can dampen their defense mechanisms to enhance symbiosis with fungi. This is achieved through internal “braking” mechanisms that suppress immune signaling pathways and the expression of defense genes [[45], [46], [47]]. On the other hand, small-secreted proteins derived from *Populus trichocarpa* can affect hyphal growth and morphology [48].

Along with mycorrhiza, fungal endophytes have emerged as a distinct group that form *mutualistic associations* with the plant, providing various benefits, including tolerance to both biotic and abiotic stressors (e.g. pathogens and environmental challenges including drought, extreme temperatures, salinity, heavy metals, and others). It has been reported that plants' recognition and recruitment of fungal endophytes is mediated by secreted chemical signals. Examples of these chemical signals include phenolic acids [49], exopolysaccharides [50], and terpenoids [51]. Once recognized, a series of signal transmission and transduction events occur, followed by the activation of the plant's defense network. Various mechanisms have been identified through which endophytes can evade and manage the plant's defense responses, eventually establishing a balanced symbiotic relationship that helps the plant withstand biotic and abiotic stresses [52]. Recently there has been an increased interest in dark septate endophytes (DSE) that have been shown to protect plants from abiotic and biotic factors [53], recruit nutrients from the environment to the host plant [54] and regulate the plant and soil microbiomes [55]. As more DSE are isolated and characterized, these groups of fungi could be the future of understanding how endophytes benefit a plant's development.

The molecular pathways involved in plant symbiosis with AMF and nodulating bacteria share a signaling pathway called the common symbiosis signaling pathway (CSSP). This pathway involves the activation of a calcium/calmodulin-dependent protein kinase (CCaMK) and its associated transcription factor, CYCLOPS [[56], [57], [58]].

In addition to plant molecular pathways underlying fungal associations, recent research highlights additional pathways contributing to beneficial bacterial associations [[60], [61], [62], [63]]. Such interactions can provide the host plant with enhanced tolerance to environmental stress, resulting in more robust yields with reduced need for water, fertilizers, and pesticides. This not only minimizes environmental impact but also drives more sustainable and cost-effective bioenergy crop production. For example, plant bacterial endophytes can enhance host thermotolerance through various means, including triggering heat-responsive genes, producing protective compounds, and influencing nutrient allocation [[64], [65], [66]]. This is particularly important as climate change brings more frequent and intense heat waves [67,68]. Under such pressure, the plant's microbial community adapts rapidly, potentially shaping the plant's ability to tolerate heat and enhance fitness [[69], [70], [71]]. It was recently demonstrated that microbes can adjust their community composition in response to heat, and that these heat-tolerant microbes can transfer heat tolerance to plants [72].

It is likely that improved plant performance under abiotic stress is the result of a combination of microbial mechanisms working together. In recent years, significant advances have been made in deciphering the plant molecular pathways involved in their symbiosis with microbes through various omic approaches. As a result, several protein components of the symbiotic signaling pathways have been identified (Fig. 2). Nevertheless, there are still missing elements in the overall picture, and several crucial points remain unclear. An essential way to comprehend the molecular genetic interactions driving critical connections involves using synthetic biology tools, such as pathway manipulation and biosensor engineering (see Sections 2.3, 3.1).

Lastly, these molecular insights will significantly enhance plant breeding efforts and management practices aimed at improving plant resilience to stresses. By harnessing knowledge of the molecular pathways involved in plant-microbe symbiosis, researchers can develop targeted breeding strategies and genetic modifications that reinforce crop adaptability to challenging environmental conditions. Additionally, this understanding can guide effective management practices (e.g., crop rotation, cover cropping, and pest management) to promote soil health and foster beneficial microbial communities.

### 2.3 Overlap between symbiotic and immune signaling pathways in plants

**Fig. 3:**
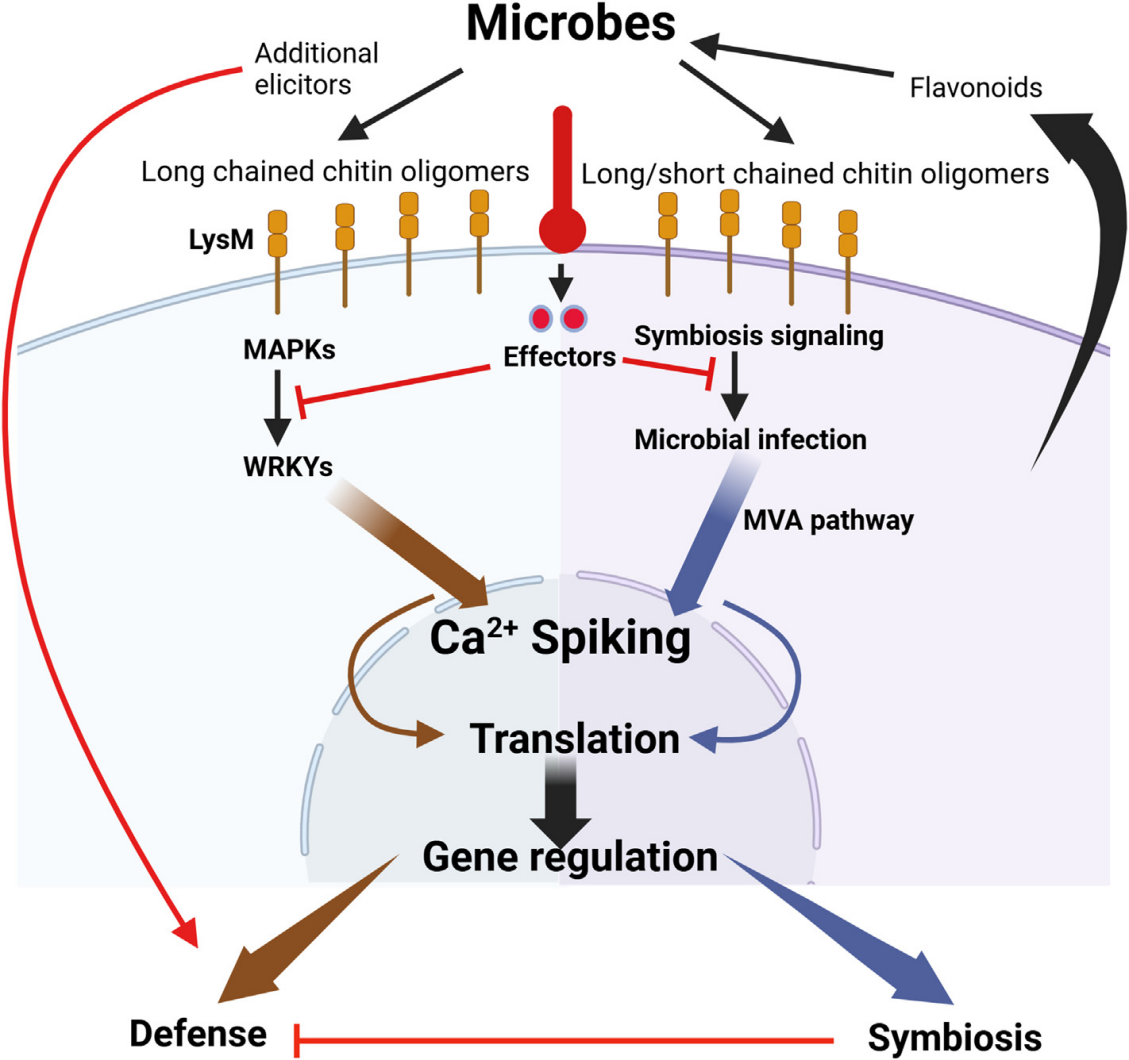
Partial overlap between symbiotic and immune signaling pathways in plants. Microbes produce long chained chitin oligomers (COs) which is recognized by LysM. LysM activates mitogen-activated protein kinases (MAPKs) that activates WRKYs resulting in calcium spiking, which regulates genes responsible for defense against pathogens. On the other hand, microbes that release long and short chained COs can also activate LysM to activate symbiosis signaling and result in microbial infection. The infection activates the Mevalonate (MVA) pathway resulting in calcium spiking and thus regulating genes involved in symbiosis with microbes. The effectors secreted by microbes affect both defense and symbiosis mechanisms. Some additional elicitors secreted by microbes can result in plant defense attenuation. Arrows indicate positive regulation and T-shape arrows indicate negative regulation. Redrawn from Refs. [73,91,92]. Created with BioRender.com.

It has been suggested that plant immunity and symbiosis signaling partially overlap at multiple levels [73], as illustrated in Fig. 3. For example, GhWAK13, a cell wall-associated Ser/Thr kinase, plays a central role in both symbiotic and immune signaling [74,75]. Also, LysM receptor-like kinase NFR1/NRF5/LYK3/LYK8/OsCERK1 represents a shared receptor for chitooligosaccharide-based signals, such as lipo-chitooligosaccharides (LCOs) and short-chain chitooligosaccharides (COs) [59,[76], [77], [78], [79], [80]]. These LysM receptors are determinants of specificity to these signaling molecules in plants [81] and LCOs and COs are derived from both beneficial microbes and fungal pathogens [82]. Although the same receptors, such as LysM kinases, can recognize signals from both beneficial microbes and pathogens, plants differentiate between the two through context-specific downstream signaling [83]. For symbionts, like mycorrhizal fungi or nitrogen-fixing bacteria, plants suppress immune responses to allow mutualistic interactions, such as nutrient exchange or root hair branching in legumes, triggered by LCOs [84]. On the other hand, when encountering pathogens, the same receptors activate immune responses to defend against infection by producing antimicrobial compounds and reinforcing the cell wall [80]. Microbial-produced LCOs have been shown to elicit root hair curling in legumes as demonstrated in *Medicago truncatula* and *Vicia sativa* [82]. Interestingly, these same LCOs also appear to influence fungal behavior and physiology, and their surrounding microbial communities by triggering the production of secondary metabolites from *Aspergillus fumigatus* and *Laccaria bicolor* [82,[85], [86], [87]], suggesting the presence of a receptor in microbes that detect these signals. In the rhizobium *Bradyrhizobium japonicum,* LCOs or COs with a tetrameric backbone exert feedback regulation on nodulation genes, which can be key components required for efficient and effective nodulation [88,89]. The growing evidence that LCOs and COs affect both LCO/CO-producing and non-producing microbes suggest a broader ecological role for these molecules. However, it remains unclear whether microbes possess receptors that perceive these signals, and if they are analogous to the LysM receptors found in plants. Based on the previous work [90], we speculate, however, that LysM-receptor-like kinases may function as cell surface pattern recognition receptors for N-acetylglucosamine molecules and their derivatives.

## 3 Application of plant synthetic biology for understanding plant-microbe interactions

**Fig. 4:**
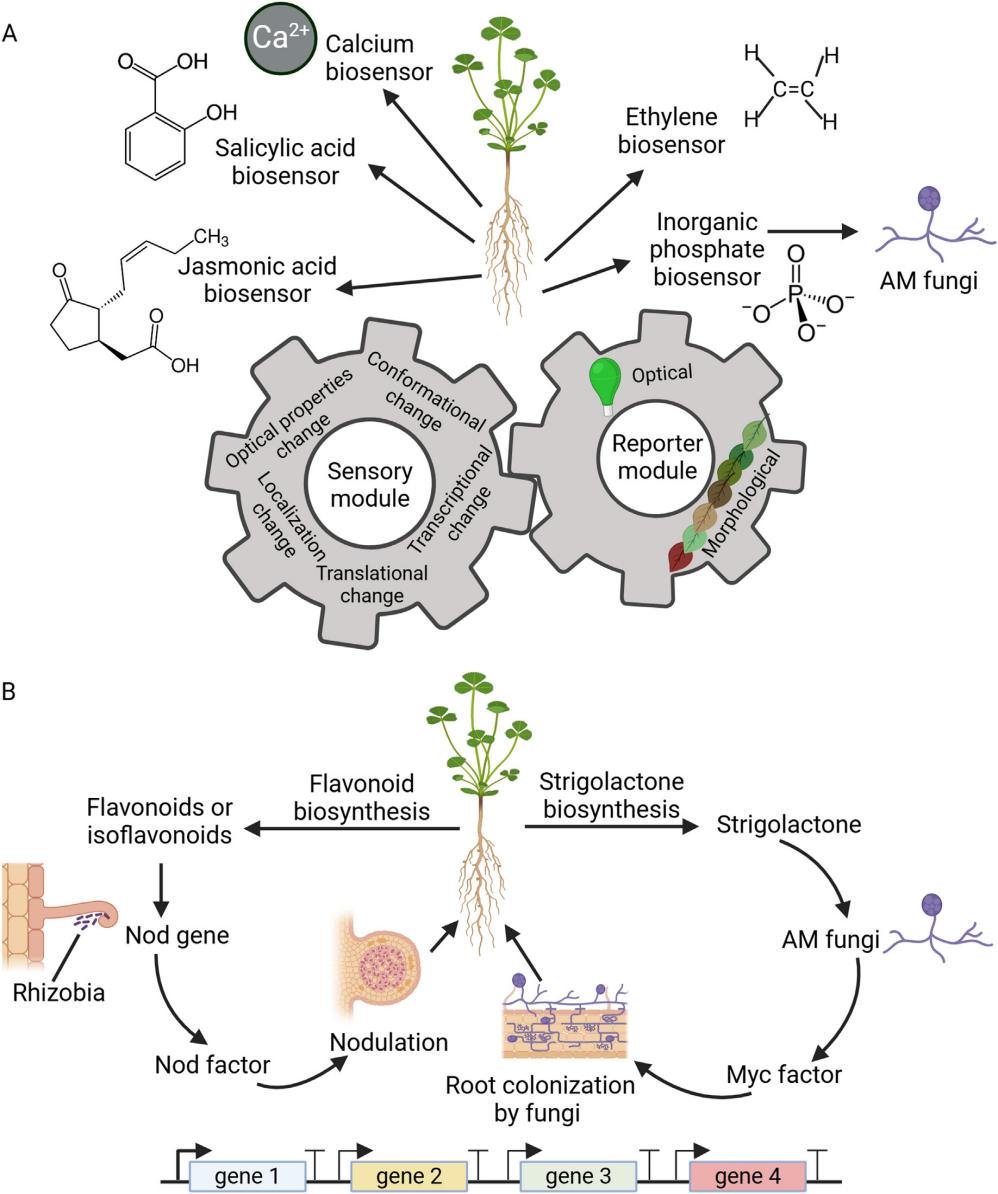
Application of plant synthetic biology for understanding plant-microbe interactions. (A) Plant based biosensor engineering for studying the molecular signaling in plant-microbe interactions. (B) Pathway engineering for dissecting the molecular interactions between plants and microbes. Redrawn from Refs. [[93], [94], [95], [96], [97]]. Created with BioRender.com.

Plant synthetic biology offers powerful tools for dissecting molecular interactions between plants and microbes, such as plant-based biosensors for studying the molecular signaling in plant response to microbes and pathway engineering for unraveling the complexity of plant molecular mechanisms underlying disease resistance or symbiosis, as illustrated in Fig. 4.

### 3.1 Engineering of plant-based biosensor for studying the molecular signaling in plant-microbe interactions

Genetically encoded plant-based biosensors (GEPBs), which repurpose the logic circuitry and biological and molecular components used by plants in nature to direct signal recognition mechanisms into carefully assembled outcomes that can be easily detected [97], have great potential for studying the molecular signaling involved in plant-microbe interactions. Calcium (Ca^2+^) serves as a vital secondary messenger linking microbial signal detection to the initiation of proper immune and/or symbiotic responses in plants [96]. Also, reactive oxygen species (ROS), such as hydrogen peroxide (H_2_O_2_), accumulate in infected plant cells in response to attacks by microbial pathogens, serving as a key signal mediating the plant adaptive response to pathogenic microbes [98,99]. GEPBs for *in vivo* imaging of Ca^2+^ and ROS have been well established [94]. For example, *Arabidopsis* plants expressing the fluorescence-based Ca^2+^ sensor R-GECO1 have been used to observe flagellin (flg22)- and chitin-induced Ca^2+^ signals, allowing for the identification of defined cytosolic calcium [Ca^2+^]_cyt_ oscillations in response to the fungal elicitor chitin [100]. To facilitate the studies of PTI responses in *Arabidopsis*, the H_2_O_2_ biosensor roGFP2-Orp1 and the glutathione redox state biosensor GRX1-roGFP2 were created to reveal oxidative events in response to flg22 and *Pseudomonas syringae* [101]. Plant hormones, such as salicylic acid, ethylene, and jasmonic acid, play central roles in interactions with pathogenic and beneficial microbes [102]. Outside of applying GEPBs to detect Ca^2+^-/ROS-mediated signaling in plant-microbial interactions, the application of plant-based biosensors in hormone-mediated signaling in plant responses to microbes remains very limited [94]. To enable *in vivo* quantitative analysis of hormone signaling output in plants in response to microbes, modular fluorescent protein-based biosensors (COLORFUL-PR1pro, -VSP2pro, and -PDF1.2apro) have been engineered in *Arabidopsis* to study cell-type specific hormone activities during microbial invasion [103].

In addition to utilizing GEPBs to detect the output from signaling mediated by Ca^2+^, ROS, and plant hormones, other aspects of plant-microbe interactions, e.g., nutrient exchange between plants and associated microbes, can be monitored by plant-derived biosensors. For example, fluorescence resonance energy transfer (FRET)-based inorganic phosphate (P_i_) biosensors have been engineered in *Brachypodium distachyon* to monitor hyphae-mediated orthophosphate transfer to mycorrhizal root cells in response to two AMF species *Diversispora epigaea* (formerly *Glomus versiforme*) and *Gigaspora gigantea* [104].

### 3.2 Pathway engineering for dissecting the molecular interactions between plants and microbes

Plants produce various metabolites encompassing plant primary metabolites, phytohormones, and plant secondary metabolites (PSMs), to shape their interactions with microbes. Pathway engineering can be employed to unravel the molecular basis of plant-microbe interactions by modifying or constructing new metabolic or signaling pathways in plants. Specifically, engineering metabolic pathways, which produce specific compounds related to defense or symbiosis, can be used to study the roles of these metabolites in plant-microbe interactions. Plant secondary metabolites, such as flavonoids, can recruit beneficial microbes like rhizobia bacteria or mycorrhizal fungi to initiate LCO production and colonization of the plant [95]. Interestingly, flavonoids can also be antimicrobial [[105], [106], [107]]. There are numerous examples of the multifaceted roles of flavonoids in plant-microbial interactions [93]. Consequently, a deeper understanding of the role that flavonoids have in attracting beneficial microbes while simultaneously deterring pathogens is needed. Once such flavonoids have been identified, -omics data (e.g., transcriptomics, metabolomics, proteomics), along with computational systems biology, can be used to predict the key genes involved in the flavonoid biosynthetic pathways to then manipulate (e.g., over-expression) the functions of these genes. Simultaneous manipulation of different combinations of the predicted candidate genes can then provide a comprehensive understanding of the metabolic pathways involved in flavonoid biosynthesis in the context of plant-microbe interactions.

Among the complex secondary metabolites that plants produce, a subset are volatile organic compounds (VOCs). Despite their enormous structural diversity, most plant VOCs belong to three chemical classes: terpenoids, phenylpropanoids/benzenoids, and fatty acid derivatives. The general pathways leading to these three classes of VOCs are well understood. However, since many genes involved in VOC biosynthesis belong to multi-member families [108], identifying specific genes responsible for the production of specific VOCs in any given species often requires experimental validation. Plant VOCs mediate a myriad of plant-environment interactions, including plant-microbe interactions. Many plant VOCs serve as chemical defenses against pathogenic microbes. For example, the volatile monoterpene (*S*)-limonene in rice defends against the blast fungus *Magnaporthe oryzae* [109], and the volatile sesquiterpene (*E,E*)-α-farnesene in soybean defends against soybean cyst nematode [110]. Some plant VOCs attract microbial allies; for example, the volatile sesquiterpene (*E*)-β-caryophyllene in maize roots attracts entomopathogenic nematodes that infect and kill root herbivores [111]. Plant VOCs can sometimes be exploited by microbial natural enemies of the host. For example, some plants produce the volatile methyl salicylate as a defense compound in the root. This volatile functions as a chemical defense against pathogenic soybean cyst nematode in soybean [112], yet in tomato, it serves as an attractant for pathogenic root-knot nematodes [113]. This reflects the dynamic arm's race of defense and counter-defense between plants and microbes. It is worth noting that microbes, especially fungi [114] and bacteria [115], also produce diverse VOCs. While VOCs from some plant growth-promoting rhizobacteria have been shown to promote the growth of *Arabidopsis* [116], the impact of microbial VOCs on host plants is poorly understood. Plant synthetic biology is poised to disentangle both the chemical diversity of plant VOCs and the complexity of the plant-microbe interactions they mediate. By enabling precise manipulation of biosynthetic pathways, engineering targeted gene expression, and developing synthetic regulatory circuits that mimic natural processes, synthetic biology approaches will facilitate the systematic exploration of VOC biosynthesis and the construction of tailored plant-microbe interaction models to elucidate underlying mechanisms.

Strigolactones (SLs) also play an important role in beneficial symbioses with arbuscular mycorrhizal fungi (AMF) [117]. Overexpression of a petunia SL transporter (PaPDR1) in *Medicago truncatula* enhances AMF colonization, highlighting the role of ABCG proteins in the SL translocation to facilitate AM symbiotic interactions in diverse plant lineages [118]. Heterologous expression of the canonical SL receptor D14 of *Arabidopsis* (*AtD14*) renders the cells of *Marchantia paleacea* (a plant species in the oldest of the extant lineages of land plants) sensitive to bryosymbiol, indicating an ancestral function of SLs as symbiotic rhizosphere signals [119]. Although the genes responsible for the early stages of SL biosynthesis are well-known in several plant species, the later stages of the pathway, where flux branching leads to the production of alternative SLs, remain less understood. Mathematical modeling suggests that engineering this pathway to modulate SL production could be most effectively achieved by increasing the flux of β-carotenes entering the biosynthetic pathway [120]. This model-guided pathway engineering strategy could provide a deep understanding of SL-mediated signaling pathway involved in the symbiosis of plants with AMF.

Other phytohormones, such as jasmonic acid (JA) and ethylene (ET), play pivotal roles in activating plant defense responses. Heterologous expression of two *Salix purpurea* G-type lectin receptor-like kinases (LecRLKs) with difference in the PAN-Apple-Nematode (PAN) domain in *Arabidopsis* revealed that G-type LecRLKs can modulate JA and ET signaling pathways via their PAN domains, thereby influencing the expression of microbe-responsive genes [121], providing a new insight into the molecular mechanism of JA/ET-mediated signaling in plant-pathogen interactions and the application of plant synthetic biology for understanding plant-microbe interactions.

## 4 Application of plant synthetic biology for increasing plant disease resistance

**Fig. 5:**
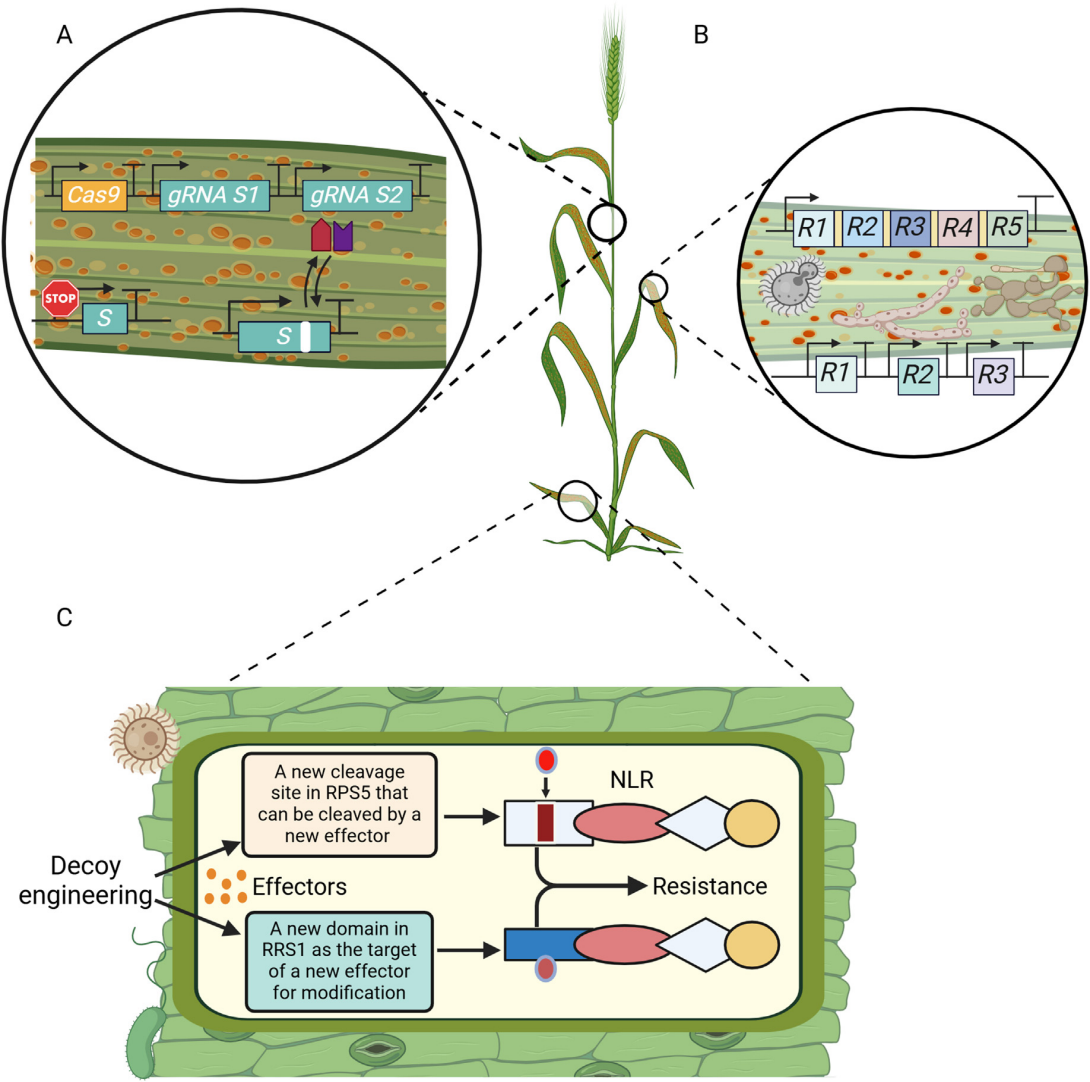
Application of plant synthetic biology for enhancing plant disease resistance. (A) Engineering Susceptible (*S*) genes using CRISPR based editing or RNAi of S-susceptible gene can induce durable and broad-spectrum resistance, promotor editing, and single nucleotide polymorphism can also induce disease resistance in wheat [126]. (B) Stacking resistance (*R*) genes can induce broad-spectrum disease resistance in wheat [125]. (C) Engineering synthetic receptors by changing cleavage site or introducing decoy domain can induce disease resistance [127]. NLR: Nucleotide-binding leucine-rich-repeat. gRNA: guide RNA. Created with BioRender.com.

Plant disease resistance can be enhanced using various synthetic biology approaches, including engineering disease resistance genes, knocking out/down susceptibility genes, engineering synthetic immune receptors, and engineering inducible defense mechanisms, as illustrated in Fig. 5.

### 4.1 Stacking disease resistance genes in plants

Various crop plants have been engineered with individual disease resistance (*R*) genes. For example, the *Rpi-blb2* gene from *Solanum bulbocastanum* was introduced into potatoes to confer resistance to late blight disease, the most important disease of potatoes worldwide [122]. Overexpression of *Xa47*, which was derived from the wild rice material G252, in the susceptible rice material JG30 provided resistance to bacterial blight in rice [123]. Since many *R* genes are semi-dominant, stacking NLRs that recognize distinct effectors should strengthen plant defense and confer a genetic benefit resulting from a requirement for multiple mutations in a pathogen to evade detection by multiple immune receptors [21]. For example, transforming a 19-kb DNA construct bearing three *R* genes (*Rpi-blb2*, *RB* and *Rpi-vnt1.1*) from wild *Solanum tuberosum* (potato) species into African highland potato varieties confers complete field resistance to late blight caused by *Phytophthora infestans* [124]. Also, engineering a 37-kb DNA cassette containing five resistance genes (*Sr22*, *Sr35*, *Sr45*, *Sr50*, and *Sr55*) into bread wheat as a single locus confers broad-spectrum resistance to the fungal rust pathogen *Puccinia graminis* f. sp. *tritici* (*Pgt*) [125].

### 4.2 Engineering synthetic immune receptors in plants

Although *NLR* genes are essential targets for engineering plant disease resistance, not all *NLR* genes are useful for crop protection because their spectrum can be limited due to the polymorphism of effector-coding genes [128,129]. This challenge can be addressed by engineering plant immune receptors to extend the disease resistance spectrum [130,131]. For example, substituting the cleavage site of the pathogen-secreted protease AvrPphB within the *Arabidopsis thaliana* protein PBS1, which is guarded by the NLR protein RPS5, with decoy cleavage sites for other pathogen proteases, can expand the recognition specificity of RPS5 and confer resistance against new pathogens [132]. Similarly, it has been proposed that molecular engineering of the integrated decoy domain (ID) of a rice NLR protein could extend its recognition spectrum to a new effector AVR-PikD in the *Nicotiana benthamiana* heterologous system [128]. Also, it was reported that the specificity and autoactivity of plant NLR proteins, Sr33 and Sr50, could be altered using a rational engineering approach combining phylogenetics, allele diversity analysis, and structural modeling [133]. Furthermore, it was recently demonstrated that made-to-order synthetic plant immune receptors could be created by using NLRs as scaffolds for nanobody (single-domain antibody fragment) fusions that bind fluorescent proteins (FPs) to trigger immune responses in the presence of the corresponding FP and consequently confer resistance against plant viruses expressing FPs [134]. In combination with machine learning based on advanced classifiers, such as Random Forest, Support Vector Machine, and Extra Trees, for analyzing large datasets of immune receptor sequences to predict new receptor sequences with desired pathogen recognition capabilities [135], this made-to-order synthetic receptor technology provides an exciting opportunity for engineering NLRs to activate resistance against any pathogen.

### 4.3 Engineering susceptibility genes and immune repressors

Although resistance (*R*) genes, especially those single dominant *R* genes that mediate specific recognition of pathogens, have been the main targets of genetic engineering to enable genetic resistance to pathogens, *R*-gene-based disease resistance is often readily overcome by rapidly evolving pathogens. This limitation can be addressed by genetically manipulating plant susceptibility (*S*) genes that pathogens exploit to facilitate their infection. By targeting *S* genes involved in pre-penetration (allowing basic compatibility) and/or post-penetration (allowing proliferation), it has been possible to engineer durable and broad-spectrum resistance against filamentous pathogens such as powdery mildew fungi [[136], [137], [138], [139]]. Also, RNAi-mediated inactivation of a *S* gene (*TaPsIPK1*) encoding a receptor-like cytoplasmic kinase in wheat confers broad-spectrum resistance to rust fungi [126].

It is essential to disrupt the immune-suppressive mechanisms employed by adapted pathogens within the host's cellular environment to achieve optimal resistance levels in the host. This can be accomplished by precisely targeting and inhibiting the activity of host immune transcriptional repressors using advanced technologies [140].

CRISPR/Cas-mediated genome editing has been successfully applied in targeted mutagenesis of susceptibility factors in multiple plants [141]. For example, CRISPR-Cas9 technology was used for simultaneous knockout of three homoeoalleles encoding MILDEW-RESISTANCE LOCUS (MLO) proteins in hexaploid bread wheat to confer heritable resistance against powdery mildew [142]. Furthermore, a wheat mutant (*Tamlo-R32*), featuring a 304-kb segment targeted deletion in the *MLO-B1* locus, was created via genome-editing and conferred strong resistance to powdery mildew, while retaining normal crop growth and yields [143]. CRISPR/Cas9-mediated genome editing was also used to downregulate the expression of the SWEET sugar transporter genes (*SWEET11*, *SWEET13* and *SWEET14*) in rice plants by editing the target sites of bacterial effectors, which results in reduced sugar levels in the apoplast where the bacterial pathogen *Xanthomonas oryzae* pv. *oryzae* resides and proliferates, leading to broad-spectrum resistance to bacterial blight [144].

## 5 Engineering synthetic symbiosis pathways

**Fig. 6:**
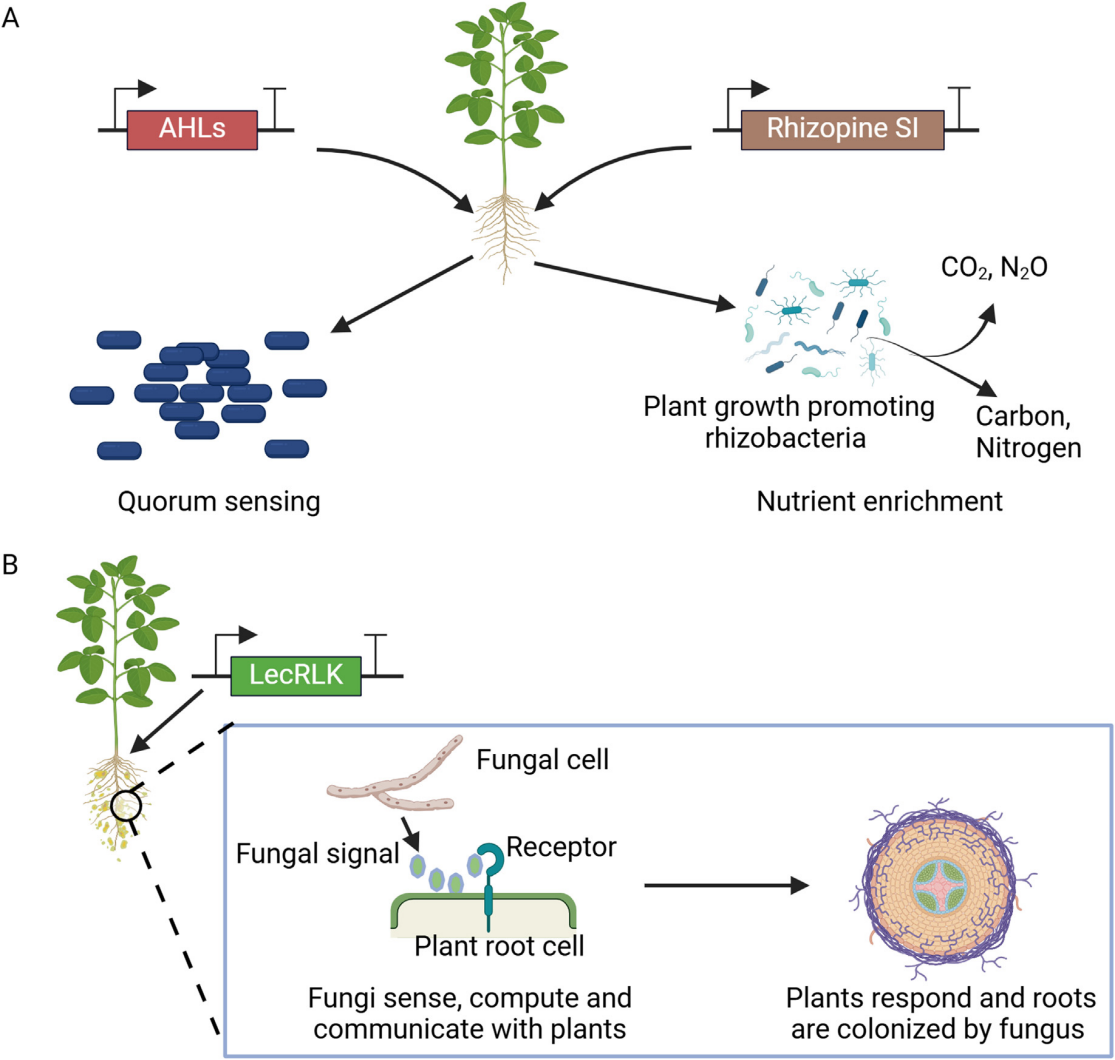
Application of plant synthetic biology for enhancing plant-microbial symbiosis. (A) Synthetic signaling genes such as N-acyl-homoserine lactones (AHLs) trigger coordinated bacterial behaviors through quorum sensing and rhizopine scyllo-inosamine (SI) and induce the expression of rhizobacterial plant growth-promoting genes which allows for targeted N_2_ fixation mediated by plant growth-promoting rhizobacteria. (B) Introduction of Receiver genes like lectin receptor-like kinase (LecRLK) mediates symbioses between plants and the ectomycorrhizal fungus. Redrawn from Refs. [145,146]. Created with BioRender.com.

Synthetic symbiosis can be established by creating genetically modified plants that produce signaling molecules that regulate the gene expression of beneficial microbes or express synthetic cell-surface receptors that bind to ligands derived from beneficial microbes (Fig. 6).

### 5.1 Engineering synthetic transkingdom signaling pathways

Plants can be engineered to produce synthetic signaling molecules to initiate interactions with their rhizosphere microbiome. One of the first attempts was to engineer tobacco plants to produce N-acyl-homoserine lactones (AHLs), a molecule critical for triggering coordinated bacterial behaviors through quorum sensing. The secretion of these AHLs by the plants was sufficient to restore the biocontrol activity of a *P. aureofaciens* strain deficient in AHL production and, inversely, restore the pathogenicity of an *E. carotovora* strain rendered avirulent through a mutation in its AHL synthase gene [147].

Plants can also be engineered to produce synthetic signaling molecules to initiate symbiotic relationships.

To create a synthetic plant-controlled symbiosis system between a desired host plant and plant growth-promoting rhizobacteria (PGPR) capable of N_2_ fixation, a synthetic pathway was engineered in *Medicago* and barley to produce rhizopine *scyllo*-inosamine (SI) [148]. SI is an inositol-derived rhizopine naturally produced by a few *Rhizobium* and *Sinorhizobium* strains housed within legume nodules during N-fixing endosymbiosis [149]. Engineering the transkingdom signal pathway in the plant host of interest and inducing the expression of rhizobacterial plant growth-promoting (PGP) genes allow for targeted PGPR-mediated N_2_ fixation. This prevents the interaction between PGPR and non-target plant species, coupling interactions in the field and preventing the growth promotion of non-target plants [146].

### 5.2 Engineering synthetic receptors in plants

Plants can be engineered to respond to synthetic signals produced by microbes. For example, based on the quorum sensing-dependent transcriptional regulator RpaR, a “receiver device” was engineered in *Arabidopsis thaliana* and *Solanum tuberosum* (potato) for detecting the small molecule p-coumaroyl-homoserine lactone (pC-HSL), which is far less abundant than AHL gleaned from bacterial quorum sensing systems, produced by engineered soil bacteria *Pseudomonas putida* KT2440 and *Klebsiella pneumoniae* 342 [145].

Plants can be engineered to express proteins on their root surfaces that serve as receptors specifically for the ligands derived from natural or engineered beneficial microbes, enabling these beneficial microbes to colonize the roots of natural non-host plants. A recent study found that a *Populus trichocarpa* lectin receptor-like kinase (PtLecRLK1) mediated the symbiosis between *Populus* and the ectomycorrhizal fungus *Laccaria bicolor* [46]. Additionally, it was demonstrated that engineering PtLecRLK1 in *Panicum virgatum*, a plant that is typically unable to be colonized by *L. bicolor*, rendered this natural non-host plant species susceptible to colonization by *L. bicolor* [47,150]. Along the same line, recent research has enhanced our understanding of Nod factor recognition by LysM receptors in legumes, paving the way for targeted *de novo* engineering in non-legumes to initiate the signaling pathway for nodule organogenesis and infection [150]. However, it is worth mentioning that engineering nodulation in non-leguminous plants may require modifications beyond just the LysM receptors. While LysM receptor-like kinases are crucial for recognizing LCOs, successful nodulation involves a complex network of signaling pathways and genetic components.

## 6 Application of plant synthetic biology for *in situ* microbiome engineering

**Fig. 7:**
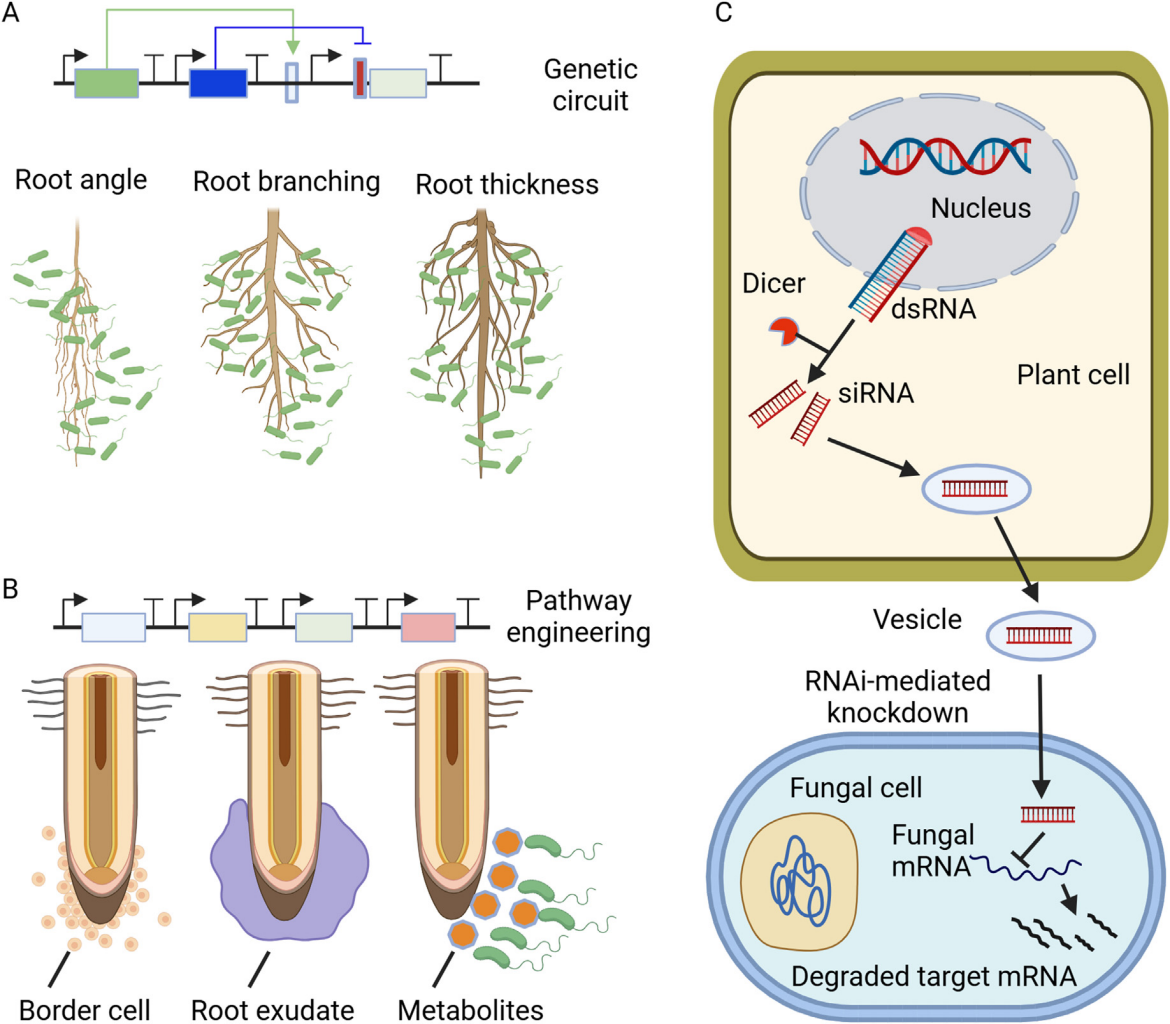
Application of plant synthetic biology for *in situ* microbiome engineering. Plant synthetic biology can be used to engineer (A) Root architecture - changing root angle, root branching and root thickness using synthetic genetic circuits to help plant microbe interaction by attracting more beneficial microbes around the roots, (B) Altering root exudate profile by pathway engineering to attract more beneficial microbes, and (C) Cross-kingdom regulation of gene expression in microbes. Redrawn from Refs. [17,157]. Created with BioRender.com.

The rhizospheric microbiome is composed of various microbes (e.g., fungi, bacteria, oomycetes, archaea), which can be either pathogenic or beneficial for plant health and fitness [41]. Using engineered plants for *in situ* microbiome engineering involves modifying plants in such a way that they directly influence and manipulate the microbial communities in their immediate environment, particularly in the rhizosphere (the soil region near plant roots). This can lead to improved plant health, growth, and productivity by fostering beneficial microbial interactions and suppressing harmful ones. This can be achieved through engineering root exudate profiles, altering root architecture, and cross-kingdom gene regulation, as illustrated in Fig. 7.

### 6.1 Engineering root exudate profiles

Root exudates play an essential role in shaping the rhizospheric microbiome by creating a favorable environment for promoting the growth of beneficial microbes over phytopathogens [151]. Plants can be engineered, using synthetic biology approaches, to release favorable root exudates (e.g., carbohydrates, flavonoids, terpenoids) for selectively promoting the growth of beneficial microbes or inhibiting pathogenic ones [41,152]. For instance, it has been shown that alterations in sucrose exudation impact solid surface motility and root colonization by *Bacillus subtilis* [153]. Furthermore, D-galactose secreted by roots can be utilized to stimulate chemotaxis and biofilm formation in *B. velezensis* [154]. Moreover, increasing the concentration of certain flavonoids in the rhizosphere enhances the mineralization of organic phosphorus by selecting microbes that produce higher levels of phosphatases, leading to improved phosphorus uptake by plants [155].

One important factor to consider is the bioavailability of root exudates under different soil conditions. For instance, a previous study demonstrated that plant-derived organic matter can inhibit flavonoids [156]. The extent of inhibition is influenced by the flavonoid's chemical structure. New tools are needed to analyze the impact of root exudation at the field scale and gain a mechanistic understanding of the interaction between signal chemistry and environmental factors to guide biodesign. Consequently, plant synthetic biology applications need to consider the profile of root exudates and the amount of secretion in relation to microbial response thresholds and ecological feedback.

### 6.2 Altering root architecture

Beyond root exudation, root architecture can play a crucial role in shaping the microbial community structure and function in the rhizosphere [158]. The characteristics of root architecture that may modify microbial communities include rooting angle and root branching [159], as well as root diameter (e.g., thin root traits, featuring limited root surface, improved root exudation, and enriched nutrient cycling, may promote bacterial diversity and activity in the rhizosphere) [158]. It was reported that the diversity of bacterial communities associated with the roots of *Robinia pseudoacacia* decreased with increasing root diameters among different root depths (0–100 ​cm) [160]. Synthetic biology has great potential for choreographing root architecture and plant-microbe interactions in the rhizosphere [17]. For example, synthetic genetic circuits, in which synthetic promoters and synthetic transcriptional regulators (including activators and repressors) are configured to form Boolean logic operations, was recently created to achieve precise spatial expression of a gene called *solitary root* (*slr-1*), which is a gain-of-function mutation in the developmental regulator *INDOLE-3-ACETIC ACID INDUCIBLE 14* (*IAA14*), in lateral root stem cells in *Arabidopsis*, resulting in the modification of lateral root branch density without impacting primary root development, root hair development, or gravitropism [18].

### 6.3 Cross-kingdom regulation of gene expression in microbes

Plant-mediated cross-kingdom regulation of gene expression in microbes can be achieved through genetic engineering of plants to produce molecules that can affect gene expression in microorganisms. MicroRNAs (miRNAs), a class of noncoding small RNAs, play a vital role in post-transcriptional gene regulation and can be transmitted between species, mediating cross-kingdom regulation by integrating specific target gene-mediated regulatory pathways [157,161]. For example, cotton plants infected with *Verticillium dahliae*, a vascular fungal pathogen causing severe wilt diseases, respond by increasing the production of microRNA 166 (miR166) and miR159, which are then exported to the fungal hyphae, inducing silencing of two fungal genes (i.e., Ca^2+^-dependent cysteine protease (Clp-1) and isotrichodermin C-15 hydroxylase (HiC-15)) [162]. Likewise, *Arabidopsis* cells can deliver small RNAs (sRNAs) via exosome-like extracellular vesicles into the fungal pathogen *Botrytis cinerea* [163]. Inspired by such natural cross-kingdom sRNA transfer from plants to associated microbes, plants can be engineered to produce synthetic RNA molecules that can be delivered into associated microbes and interfere with their gene expression through RNA interference (RNAi) mechanisms. This approach, known as host-induced gene silencing, has been demonstrated in various pathosystems. For example, expressing sRNAs, which target *Dicer-like protein 1* (*Bc-DCL1*) and *Bc-DCL2*, in *Arabidopsis* and tomato silences the *Bc-DCL* genes in the fungal pathogen *B. cinerea* [164]. Additionally, plants may secrete small proteins regulating microbe's growth and morphology. For example, *Populus trichocarpa* small, secreted proteins (PtSSPs) can enter, via *in vitro* feeding, *Lacarria bicolor* hyphae [48], and overexpression of PtSSP1 (Potri.009G063200) in the hybrid poplar clone INRA 717-1B4 (*P. tremula* ​× ​*P. alba* clone INRA 717-1B4) could enhance the ectomycorrhizal root tip formation, but inhibit *in planta* fungal growth when inoculated with *L. bicolor* [165].

## 7 Conclusion and perspectives

Exciting progress has been made in plant synthetic biology approaches for both dissecting the complexity of plant molecular responses to both pathogenic and beneficial microbes and enhancing plant disease resistance/plant-microbial symbiosis. However, there are some challenges in the application of plant synthetic biology in plant-microbe interactions research. In this section, we discuss these challenges and potential solutions. In addition, opportunities for the utilization of new plant synthetic toolboxes in plant-microbe interactions research are addressed.

### 7.1 Challenges, risks, and potential solutions for the application of plant synthetic biology in plant-microbe interactions

Plant synthetic biology has the potential to meaningfully advance research on plant-microbe interactions thus increasing agricultural sustainability. However, these applications come with several challenges and risks that must be addressed, including 1) ensuring the biosafety and biosecurity of genome-modified plants and their associated microbes, 2) maintaining the genetic stability of synthetic constructs in plants over multiple generations, 3) the specificity of plant synthetic design, to prevent unwanted, off-target effects. In addition, enhancing plant disease resistance through overexpression of resistance genes often results in adverse developmental affects in plants [166], and 4) the lack of uniform regulation of GMOs worldwide, which complicates the development and commercialization of genetically modified crops.

There has been a long-standing concern about the risk of transgene flow from genetically modified plants to compatible wild relatives. One solution to this challenge is plastid transformation, which impedes gene flow due to the maternal inheritance of the plastome. A recent report showed that plastid co-expression of two pathogenesis-related (PR) proteins (AP24 and β-1,3-glucanase) resulted in enhanced resistance against multiple pathogens, including *Rhizoctonia solani* and, *Peronospora hyoscyami* f.sp. *tabacina* and *Phytophthora nicotianae* [167]. Other methods for transgene bioconfinement include male sterility, transgene excision, and delayed flowering [168].

Genetic modification of plant hosts for *in situ* microbiome engineering may generate some unintended ecological ramifications on the resident rhizosphere microbiota due to limited understanding of the genetic mechanisms in plants that govern microbial recruitment, along with unforeseen trade-offs in plant fitness or unintended off-target effects due to altered microbiota [169]. This challenge can be potentially addressed by designing synthetic plant-to-microbe molecular communication to precisely control specific microbial targets.

It is also possible that the transgenes in genetically modified plants may be lost due to segregation or transgene silencing after multiple generations [[170], [171], [172]]. This challenge can be addressed by avoidance of repetitive sequences or multiple copies of the transgene [172], transgene insulation using insulators (e.g., the matrix or scaffold attachment regions (MARs/SARs)) [173], and the engineering of multiple genes into a single locus using a multi-gene cassette or targeted transgene integration into predetermined locations [174,175].

Lastly, the different regulatory frameworks across countries can create hurdles for global collaboration, market access, and public acceptance of plant synthetic biology innovations. To overcome this challenge, several strategies can be pursued, such as developing standardized, science-based risk assessment approaches for GMOs and helping reduce discrepancies between regulatory bodies. Establishing mutual recognition agreements on GMO safety assessments and approvals can also facilitate global collaboration. Another solution involves increasing public engagement and awareness to improve understanding of plant synthetic biology and GMOs, thereby reducing societal resistance and enhancing public acceptance.

### 7.2 Opportunities for the application of plant synthetic biology in plant-microbe interactions research

There has been rapid progress in the development of plant synthetic biology tools over the past decade, such as site-specific genome integration in plants using integrases [176], optimized transcriptional control of gene circuits using synthetic transcriptional factors (sTFs) [18], synthetic signal transduction systems [177], post-translational control mediated by split intein systems [178], programmable CRISPRi-based circuits for sophisticated spatiotemporal control of gene expression in plants [179], promoters for high-precision bioengineering [180,181], and new technologies for plant genome editing [182], along with new achievements in the development of hardware, software, and wetware codesign environment for synthetic biology [183]. These new advancements provide emerging synthetic biology approaches that permit us to answer the following critical questions concerning the interactions between plants and microbes:

• Is it possible to create a universal defense system in plants for targeted protection against major pathogens of different phyla or even kingdoms?
• Can we decipher the complexity of plant immune response in the presence of beneficial or pathogenic microbes?
• Can we identify specific root exudates or VOCs that act as chemoattractants or repellents, and thus tune host metabolism and transporters to change secretion profiles or add new pathways for rare metabolite production to manipulate community establishment, persistence, and functional impact?
• Can we engineer specific plant tissues (e.g., roots) to sense, uptake or produce specific microbial metabolites to enhance communication and resource exchange with its microbiome?
• Can we spatially and temporally control plant-microbe communications to improve ecosystem health and resilience?
• Can we engineer new orthogonal communication channels for long-distance interactions between plants and microbes?
• To what extent can precision engineering (e.g., cell-type specific genomic editing) be applied to modify plant pathways relevant to plant-microbe interactions?

**Fig. 8:**
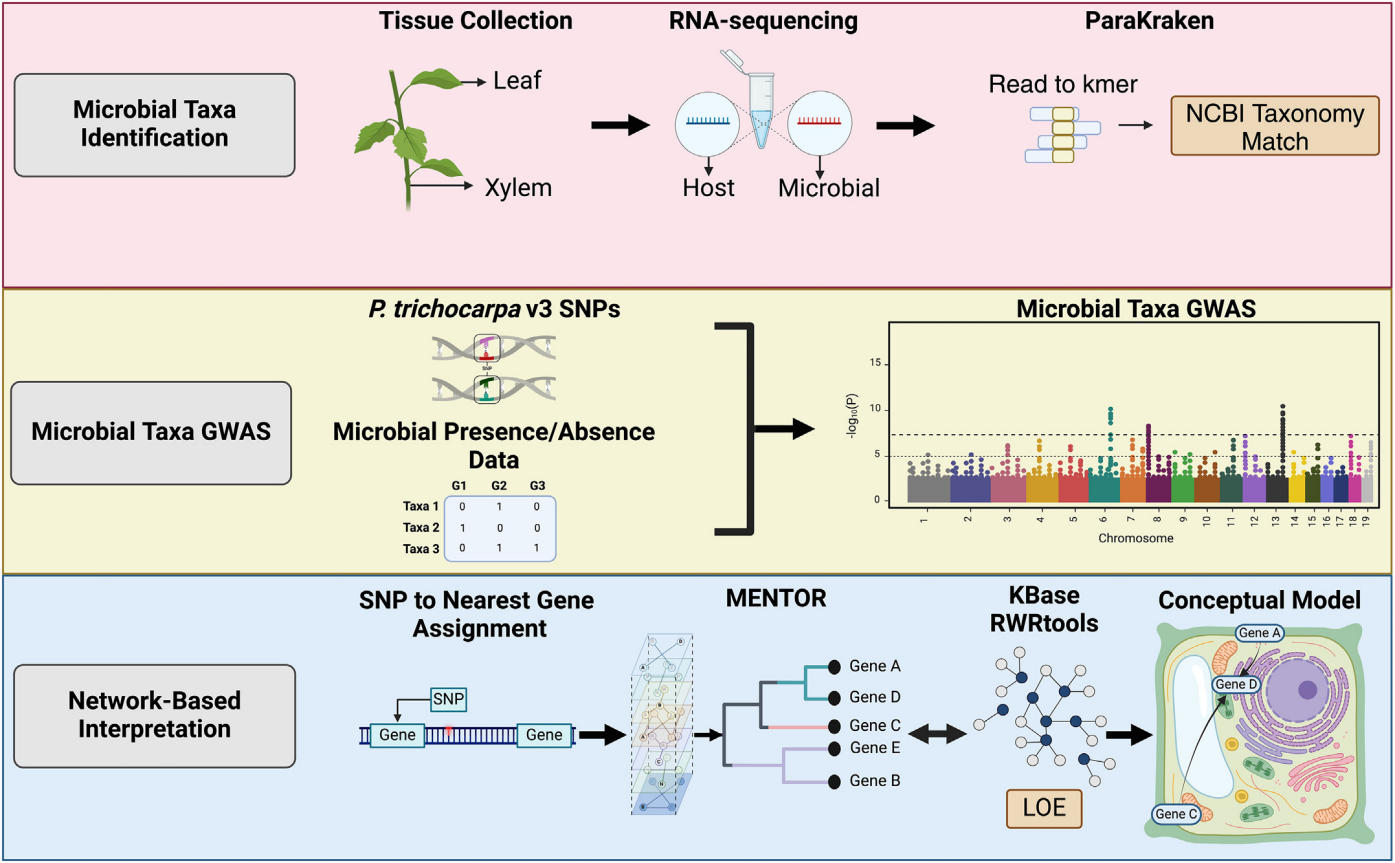
An integrative systems biology workflow demonstrating the use of genomic and network-based approaches to dissect plant-microbe interactions. Microbial taxa associated with diverse plant genotypes are identified and used as phenotypic traits in genome-wide association studies (GWAS). These GWAS analyses are conducted across microbial species associated with the plant genotypes, allowing for the identification of genetic loci involved in microbial association. The results are further integrated with differential omics datasets (e.g., transcriptomics, metabolomics) to provide a comprehensive view of plant-microbe interaction dynamics. Leveraging multiplex network methods like MENTOR (Multiplex Embedding of Networks for Team-Based Omics Research) and LOE (Lines of Evidence), this approach enables the identification of combinatorial genetic mechanisms that influence plant cellular responses, promoting the colonization of beneficial microbes or suppressing pathogens. The workflow demonstrates the effectiveness of integrating GWAS with advanced network methodologies for understanding complex plant-microbiome interactions. Created with BioRender.com.

Rapid advancements in computational biology bring more and more opportunities for the application of plant synthetic biology in plant-microbe interaction research. AI-aided integrative analysis of multi-omics and GWAS data will generate many candidate gene modules and gene networks as invaluable source information for designing multigene constructs to unravel the complexity of plant signaling/metabolic pathways responsive to microbes. Large amounts of various types of publicly available biological data, such as protein-protein interactions, predictive expression networks [184,185], and genetic associations, can be integrated into comprehensive multiplex networks. By using multiplex networks to analyze gene sets from novel GWAS and/or omics experiments, such methods (e.g., Multiplex Embedding of Networks for Team-Based Omics Research (MENTOR) [186] and lines of evidence (LOE) [187] can identify clusters of genes that are functionally interacting and/or co-regulated, providing insights into complex biological processes. The intuitive dendrogram visualization helps researchers easily interpret these relationships, highlighting key interactions, pathways and host mechanisms that are controlling plant-microbe interfaces. This approach allows for the identification of pivotal genes and pathways that play crucial roles in the plant's defense. By facilitating the interpretation of these complex datasets and promoting collaborative discussion within research teams, approaches such as MENTOR can streamline the analysis and integration of GWAS and omics datasets (Fig. 8). This enhanced understanding of gene relationships and biological pathways can lead to more targeted and effective strategies for managing plant health and improving resistance to microbial pathogens.

## Author contributions

X.Y. and J.C. conceived the idea. X.Y. T.A.R., J.T., I.D.V., S.X., D.J.W., K.D., D.J., M.M., F.C., and J.H.L. drafted the manuscript. B.M., T.A.R., J.T., M.T.I., D.J., M.M., and Y.L. designed the figures. G.A.T., T.J.T., M.J.D., J.-G. C., and P.E.A. revised the manuscript. All authors have read and contributed to the content, edited, and reviewed it.

## Funding

The writing of this manuscript was supported by the 10.13039/100000015U.S. Department of Energy (DOE) Genomic Science Program, as part of the Plant-Microbe Interfaces (PMI) Scientific Focus Area (under FWP ERKP730) and the Secure Ecosystem Engineering and Design (SEED) Scientific Focus Area (under FWP ERKPA17), and the 10.13039/100014456Center for Bioenergy Innovation (CBI; under FWP ERKP886), a DOE Research Center supported by the 10.13039/100006206Biological and Environmental Research (BER) program. Oak Ridge National Laboratory is managed by UT-Battelle, LLC for the U.S. DOE under Contract Number DE-AC05-00OR22725. This work was also supported by the National Science Foundation Plant Genome Research Program (award no. IOS-2224203) to S.X., and a SPRINT award from the 10.13039/100007135University of Tennessee, Institute of Agriculture to F.C. and J.T.

## Declaration of competing interests

The authors declare that they have no competing interests.
